# *In silico* metatranscriptomic approach for tracking biofilm-related effectors in dairies and its importance for improving food safety

**DOI:** 10.3389/fmicb.2022.928480

**Published:** 2022-08-25

**Authors:** Otávio Guilherme Gonçalves de Almeida, Marita Gimenez Pereira, Virginie Oxaran, Elaine Cristina Pereira De Martinis, Virgínia Farias Alves

**Affiliations:** ^1^Faculdade de Ciências Farmacêuticas de Ribeirão Preto, Universidade de São Paulo, Ribeirão Preto, SP, Brazil; ^2^Department of Biological Sciences, University of Texas, El Paso, El Paso, TX, United States; ^3^Faculdade de Farmácia, Universidade Federal de Goiás, Goiânia, GO, Brazil

**Keywords:** metatranscriptomic data, biofilm effectors, gene expression, dairy, milk

## Abstract

Sessile microorganisms are usually recalcitrant to antimicrobial treatments, and it is possible that finding biofilm-related effectors in metatranscriptomics datasets helps to understand mechanisms for bacterial persistence in diverse environments, by revealing protein-encoding genes that are expressed *in situ*. For this research, selected dairy-associated metatranscriptomics bioprojects were downloaded from the public databases JGI GOLD and NCBI (eight milk and 45 cheese samples), to screen for sequences encoding biofilm-related effectors. Based on the literature, the selected genetic determinants were related to adhesins, BAP, flagellum-related, intraspecific QS (AHL, HK, and RR), interspecific QS (LuxS), and QQ (AHL-acylases, AHL-lactonases). To search for the mRNA sequences encoding for those effector proteins, a custom database was built from UniprotKB, yielding 1,154,446 de-replicated sequences that were indexed in DIAMOND for alignment. The results revealed that in all the dairy-associated metatranscriptomic datasets obtained, there were reads assigned to genes involved with flagella, adhesion, and QS/QQ, but BAP-reads were found only for milk. Significant Pearson correlations (*p* < 0.05) were observed for transcripts encoding for flagella, RR, histidine kinases, adhesins, and *LuxS*, although no other significant correlations were found. In conclusion, the rationale used in this study was useful to demonstrate the presence of biofilm-associated effectors in metatranscriptomics datasets, pointing out to possible regulatory mechanisms in action in dairy-related biofilms, which could be targeted in the future to improve food safety.

## Introduction

Dairy foods are of major economic importance, and it is challenging to obtain products with high standards of safety and quality, especially due to the many processing steps from milking to serving that can be vulnerable to contamination by diverse hazards. There is great concern about microbial biofilms as reservoirs of spoilage and pathogenic microorganisms with the potential to contaminate dairies, although biofilms may also have technological importance in food processing, for example as a source of lactic acid bacteria (LAB) in vat fermentation of cheeses (Winkelströter et al., 2014; Gaglio et al., 2016; Cruciata et al., 2018). According to Marchand et al. (2012) different surfaces in dairy plants are prone to biofilm formation, including storage tanks, transport pipes, and processing equipment, especially if they are conditioned by the presence of food soil.

Biofilms can be defined as bacterial populations enclosed in a self-produced matrix, adherent to each other and/or to surfaces or interfaces (Costerton et al., 1995). The biofilm matrix is composed of extracellular polymeric substances (EPS) that may protect the cells from antimicrobial treatments (Beitelshees et al., 2018; Giaouris et al., 2015). Moreover, the cellular proximity within biofilms facilitates the exchange of genetic materials, as well as bacterial communication (Quorum sensing—QS and/or quorum quenching—QQ), with possible influence on the expression of virulence factors and regulation of the biofilm life cycle (Donlan, 2002; Utari et al., 2017; Paluch et al., 2020; Warrier et al., 2021). It has also been postulated that bacterial growth in biofilms may increase virulence, taking into account surface proteins that mediate bacterial attachment may also be involved in bacterial infection (Azizi et al., 2016; Beitelshees et al., 2018; VFDB, 2022).

Biofilms within the dairy industry may harbor important spoilage microorganisms (*Pseudomonas* sp., *Serratia* sp., LAB, thermo-resistant streptococci, and spore-forming bacteria), which can produce extracellular enzymes and other detrimental metabolites (Marchand et al., 2012; Teh et al., 2012). It has also been reported the presence of pioneer microorganisms in biofilms may facilitate the attachment of pathogens, such as *Staphylococcus aureus*, *Listeria monocytogenes*, *Bacillus cereus,* and *Campylobacter* sp. (Møretrø and Langsrud, 2017).

Effector genes codify effector proteins, which are activators or repressors acting at transcriptional or post-transcriptional levels (Wolska et al., 2016). In recent years, the molecular bases for biofilm formation have been extensively studied and it has been shown both biofilm formation and dispersal are tightly regulated by modulation of gene expression, although the effectors and the mechanisms involved in these processes are still largely unknown. From previous studies (Latasa et al., 2006) it is possible to hypothesize that even unrelated bacteria share common regulatory genes for biofilm development.

It is important to note that the majority of studies on biofilms were done with classical methods using single bacterial cultures grown in controlled laboratory conditions, in contrast to what is observed for biofilms in nature, where multiple microbial species co-exist in a complex structure, with regulatory genes likely being shared among biofilm residents (Latasa et al., 2006; Giaouris et al., 2015; Muhammad et al., 2020). Moreover, fastidious and viable but non-culturable microorganisms may fail to grow under standard laboratory conditions, which reveals the importance of applying culture-independent techniques to unravel the complexity of microbial communities in biofilms.

Metataxonomics based on *16S rRNA* and on *18S rRNA* or *ITS* amplicons have been very helpful to assess the microbial diversity of biofilms, which can be combined with metagenomics approaches (sequencing of all the genes and genomes from a given microbiome) to unravel putative metabolic pathways of the microbiota (De Filippis et al., 2016; Monnet et al., 2016; Duru et al., 2018; Liu et al., 2020; McHugh et al., 2020). To have a deeper understanding of the regulatory mechanisms in biofilm communities, studies using high-throughput cDNA sequencing can be helpful, in order to confirm functional predictions previously made from DNA analysis.

Based on the hypothesis that metatranscriptome from dairy-associated samples can reveal putative effector genes involved in biofilm formation, this paper presents an *in silico* research carried out with selected gene markers from publicly available metatranscriptomics data from milk and dairy samples, in order to contribute for improving biofilm control.

## Materials and methods

All the publicly available metatranscriptomes originated from dairy products presenting metadata described on the Joint Genome Institute Genomes Online Database (JGI GOLD)^1^ and/or at the National Center for Biotechnology Information (NCBI)^2^ were selected and downloaded on January 2021 for further downstream analysis. The keywords used in the search were “dairy,” “butter,” “milk,” “yogurt,” “cheese,” and “cream.” The term “biofilm” was not included in the keywords, since a preliminary survey revealed there was no metatranscriptomic data available in any of the searched databases regarding dairy food-associated studies on biofilms. Only the metatranscriptomics data were chosen in order to get a snapshot of the active fraction of the community with the potential to form biofilms, as indicated by the presence of selected mRNA. The searches on the databases returned only four milk and cheese-associated bioprojects (Gs0150357, Gs0117939, PRJEB23938, and Gs014492). A total of 53 samples were analyzed, comprising milk samples deposited from the Netherlands (*n* = 8) and cheese-associated samples from Italy (*n* = 27), France (*n* = 12), and Finland (*n* = 6).

A customized database comprising biofilm-related effectors was built according to validated gene categories found in the literature for adhesins, biofilm-associated protein (BAP), flagellum-related, and QS/QQ. Taking into account this latter category is highly heterogeneous due to different mechanisms of bacterial communication, the QS effectors and antagonists were further subdivided, as follows: N-acyl-homoserine-lactone (AHL), autoinducers (AI), AHL-acylases, and AHL-lactonases (Wang et al., 2019; Paluch et al., 2020). Besides, a similar approach was followed for the analysis of two-component systems (TCS) involving auto-inducing peptides (AIP), based mainly on histidine protein kinases (HK) and response regulator proteins (RR). The former catalyzes autophosphorylation at a conserved histidine residue and often possesses phosphatase activity toward cognate phosphorylated RR (Gao et al., 2007; McLoon et al., 2011). The RR comprise a major family of signaling proteins in prokaryotes, with a modular architecture consisting of a conserved receiver domain and a variable effector domain, which allows RR to function as phosphorylation-regulated switches that couple a wide variety of cellular behaviors to environmental cues, including biofilm formation (Gao et al., 2007). To identify unequivocal genes encoding these proteins, all the RR genes families described were consulted on the Prokaryotic 2-Component Systems database (P2CS, http://www.p2cs.org/), and 35 genes were retrieved: *AlpA*, *Amir*, *ArsR*, *BetR*, *CheA*, *CheB*, *CheC*,*CheV*, *CheY*, *CsrA*, *FrzZ*, *HxlR*, *LmbE*, *LysR*, *LytTR*, *MerR*, *NarL*, *NasR*, *NtrC*, *ompR*, *PglZ*, *PilB*, *PleD*, *PrrA, RpfG*, *RsbU*, *Sarp*, *Spo0A*, *TrxB, VieA*, *VieB*, *WcaA*, *XRE*, *YcbB,* and *YesN.*

All the proteins coded by these gene effectors were downloaded from the UniprotKB database using the script available at: https://github.com/Otavio20/General_scripts, summing 1,947,563 sequences. These protein sequences were de-replicated using the cd-hit tool version 4.7 with the parameter “-c 0.97” to bin sequences with 97% of global sequence similarity in the same cluster to avoid redundancy and to save computational memory resources. The non-redundant database resulted in 1,154,446 de-replicated sequences and it was indexed using DIAMOND for blasting purposes (Buchfink et al., 2015).

The bioinformatic analysis was performed by cleaning the raw sequence data downloaded from the NCBI using the bbduk tool (Bushnell et al., 2017) to remove adapters and sequences with lower-quality scores (Phred values lower than 30).

The filtered sequences were then blasted, using Diamond blastx parameters, against the custom database. Only the aligned sequences presenting at least 95% of sequence identity and e-values of 0.001 were considered. The alignments were parsed using custom Python scripts and summarized in a counting table for visualization on the R package ggplot2.

From the counting table data referring to the six main categories of biofilm-associated effectors, a Pearson statistical test was performed using the R package corrplot, considering as significant associations only those with *p*-values lower than 0.05.

## Results

**Figure 1:**
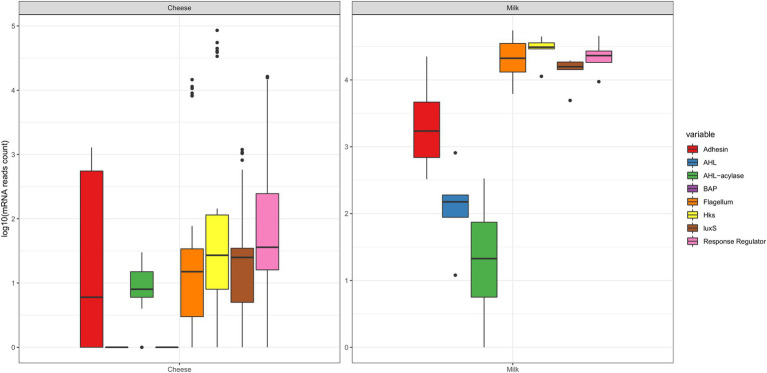
Boxplots showing the abundance variation of biofilm-related effectors expressed by the microbiota from cheese and milk samples (log_10_ read counts of mRNA), according to search done in publicly available metatranscriptomic databases, which included: adhesin, BAP, flagellum-related, intraspecific QS (AHL, HK, and RR), interspecific QS (LuxS) and QQ (AHL-acylases). The horizontal lines inside the boxes indicate the median values, and the dots outside the boxes represent outliers. Lines were plotted for the AHL and BAP in the cheese group because singletons were detected. BAP reads were not detected for milk samples. The RR category comprises a great diversity of effectors, and they presented large expressions (as detailed next in Figure 2).

**Figure 2:**
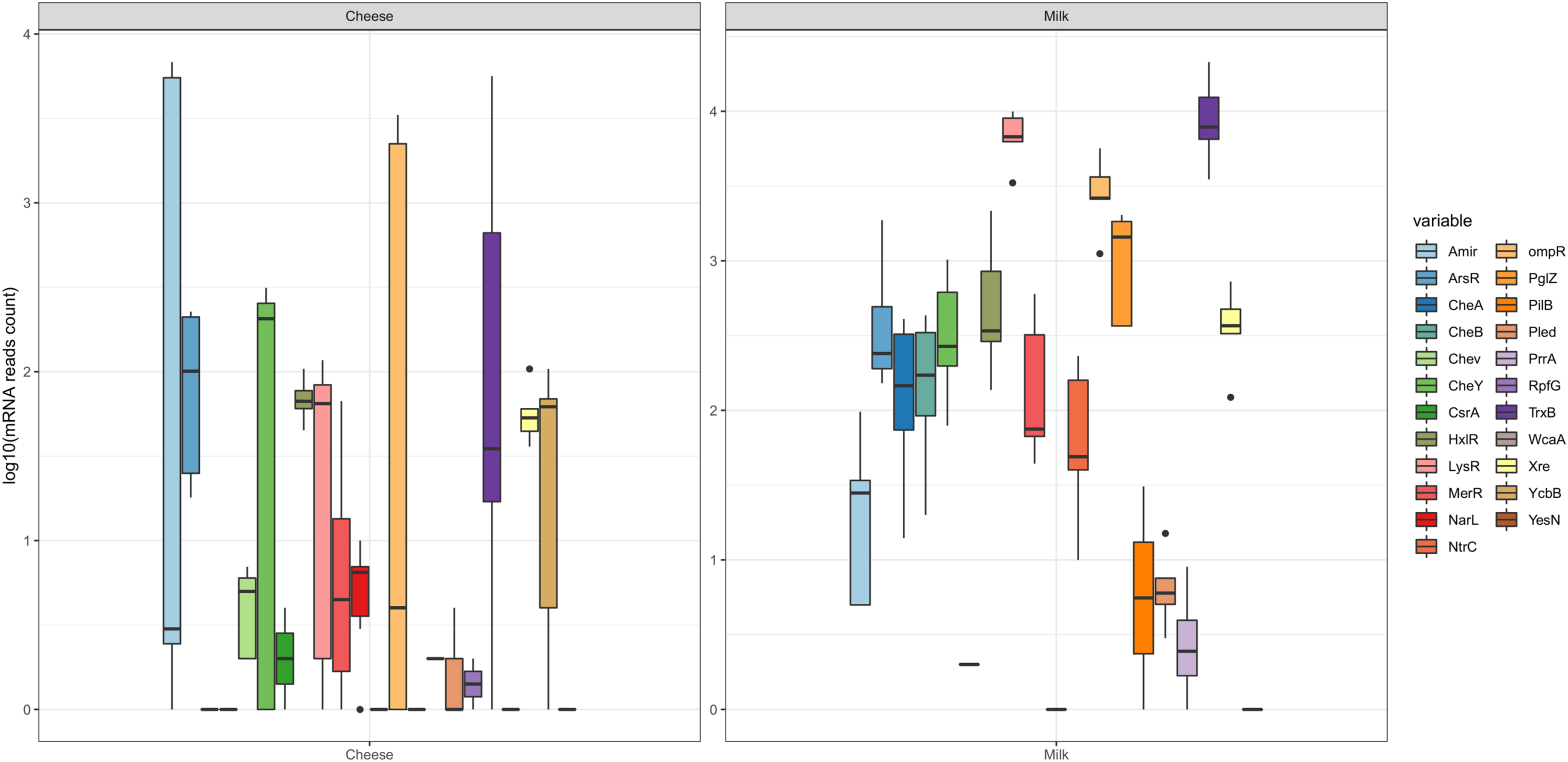
Boxplots detailing the variation of gene expression (log_10_ read counts of mRNA) for the 23 gene families of Response Regulators (*Amir, ArsR,CheA, CheB, CheC,CheV, CheY, CsrA, HxlR, LysR, MerR, NarL, NtrC, ompR*, *PglZ, PilB*, *PleD, PrrA, RpfG, TrxB, XRE, WcaA, YcbB*, and *YesN*) found in publicly available metatranscriptomic databases for the microbiota of dairy products (milk and cheeses). The horizontal lines inside the boxes indicate the median values, and the dots outside the boxes indicate outliers. Reads from single samples (lines) were obtained in cheeses for the effectors *CheA*, *NtrC*, *PglZ*, *PilB*, *WcaA*, and *YesN*, and there was no detection of *PrrA.* In milk, single lines were obtained for *CsrA, NarL* with no detection *of CheV, RpfG, Wcaa,* and *YesN*.

The boxplots from Figure 1 represent the abundance of transcripts per sample (log_10_ mRNA reads) considering the main categories of effectors surveyed in this study: adhesins, BAP, flagellum-related, intraspecific QS (AHL, HK, and RR), interspecific QS (LuxS) and QQ (AHL-acylases, AHL-lactonases). It can be observed that all transcripts were detected in milk samples (except for BAP), which indicates the large potential of the milk microbiota to form biofilms. On the other hand, in datasets from cheese samples, transcripts of adhesin genes were the most abundant, compared to transcripts from genes encoding for AHL and BAP. Figure 1 also shows that the RR category of intraspecific QS was very diverse, and thus, it was studied in more detail (Figure 2), revealing the presence of 23 out of the 35 gene families that had been searched. More specifically, for RR regulators with a DNA-binding effector domain, the gene families detected were *Amir, ArsR,CsrA, HxlR, LysR, MerR, NarL, NtrC, ompR*, *PrrA*, *XRE, YcbB,* and *YesN.* RR that present an enzymatic effector domain were also detected: *CheA, CheB, CheC, CheY, PglZ, PilB*, *PleD, RpfG, TrxB, and WcaA*, besides *CheV*, which is a regulator displaying a protein binding domain. Moreover, it was noted the greatest variations for RR occurred in the cheese samples, considering the reads from the gene families *Amir*, *CheY*, *LysR*, *ompR,* and *TrxB*.

**Figure 3:**
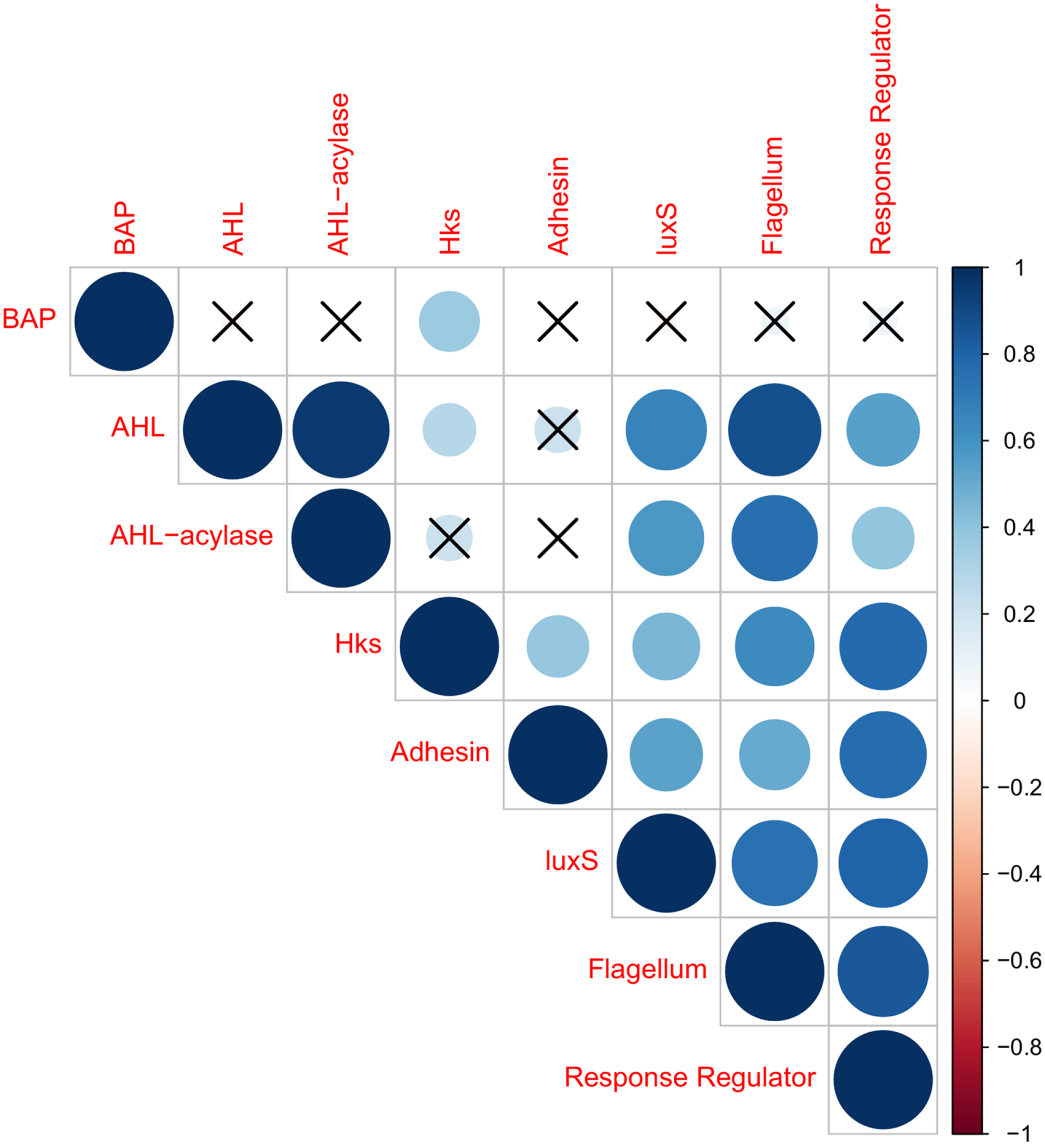
Correlation coefficient matrix for putative biofilm-effectors in samples from cheeses and milk, plotted as an upper triangle graphic, showing the correlation coefficients as circles (Pearson correlation). Insignificant markers (X) indicate which pairs of variables are not significantly related (value of *p* > 0.05). The stronger the correlation is, the larger the circle. Positive correlations are displayed in blue, while negative correlations would be displayed in red. The effectors studied were: adhesin, BAP, flagellum-related, intraspecific QS (AHL, HK, and RR), interspecific QS (LuxS), and QQ (AHL-acylases).

With regard to the correlations among the classes of effectors, the results of Pearson’s correlation analyses are shown in Figure 3, considering only significant associations (*p* < 0.05). It was observed that BAP had no correlation with any other effector, except for a weak correlation with HK. It is interesting to note AHL was strongly correlated with AHL-acylases, which might indicate the presence of antagonism among members of the dairy microbiota. AHL presented also a strong correlation with flagellum-related effector, and weaker correlations with LuxS, RR, and HK. Moreover, AHL had no correlation with adhesin. On the other hand, AHL-acylases were strongly correlated with flagellum-related effector, presenting weaker correlations with LuxS and RR. Moreover, there was no correlation of HK and adhesins. Significant numbers of HK reads were correlated with the RR, which is a consistent result considering these two effectors might act in synergism. The HK reads were also correlated with flagellum-related effectors, luxS, and adhesin, in decreasing magnitude. Reads from the gene encoding for adhesin presented a strong correlation with RR effectors, and it correlated less intensely with luxS and flagellum-related effectors. LuxS presented a high correlation with flagellum-related and RR reads. Flagellum-related effector presented a high correlation with RR. Conversely, negative correlations were not observed for any of the effectors studied in milk and cheese microbiota.

## Discussion

Studies on transcriptomics of dairy-associated microorganisms are relatively scarce, and no public metatranscriptomics datasets on biofilms from the microbiota of milk and dairy samples were available. However, it is relevant to find effectors that are potentially involved in biofilm formation among the microbiota of milk and dairies, since they can indicate its ability to cause persistent contamination in processing facilities. The bioprojects used in this research were not originally designed for the study of biofilms, but they were rather focused on taxonomic and functional aspects of the microbiota important for food safety and quality, with publications on those topics. It must be mentioned though that for the milk bioproject (bioproject #Gs0150357) from the Netherlands, to the best of our knowledge, there was no indexed publication with transcriptomics for the dataset, and the register of JGI database informed the objective of the bioproject was to reveal the link between species composition and functionality of microbial systems in spontaneously fermented milk.

A set of metatranscriptomic data analyzed in the present study (bioproject # Gs014492) was generated by Monnet et al. (2016) in France, with the aim of investigating the activity of specific microorganisms in the production of a surface-ripened cheese, focusing on the ability of the microbiota to use diverse substrates for energy generation. In that research, *Streptococcus thermophilus* and *Lactobacillus delbrueckii* ssp. *bulgaricus* were used for cheese preparation, with *Brevibacterium auratiacum* added for ripening. RNA was extracted from cheese rinds, and metatranscriptomes with good homogeneity were generated. Interestingly, Monnet et al. (2016) observed there were more reads derived from *Lactobacillus casei* (which was not added) than reads of *B. auratiacum,* indicating the importance of the resident microbiota for cheese ripening. The search for biofilm effectors in cheese transcriptomes carried out in this *in silico* study, indicated the potential of the microbiota for biofilm formation, which is relevant to determine the autochthonous microbiota in food processing environments.

Another bioproject (#PRJEB23938) included in the present research originated from a Finnish study on Maasdam cheese during ripening in warm and cold rooms (Duru et al., 2018). Those cheeses were prepared with two strains of *Lactococcus lactis* (primary acidifiers) plus *Propionibacterium freudenreichii*, *Lactobacillus rhamnosus,* and *Lactobacillus helveticus* (adjunct cultures). Based on metagenomic datasets, Duru et al. (2018) reconstructed the genomes of *L. lactis*, *L. rhamnosus, L. helveticus,* and *P. freudenreichii* subsp. *shermanii* strain JS from the Swiss-type cheese, reporting a strong dominance of *L. lactis* within the microbial community (~80%–90%), with low abundance of DNA reads from milk environmental bacteria. At the same time, RNA-seq mapping to genome bins reinforced the metagenomic findings on the dominance of *L. lactis*, with gene enrichment analysis indicating the presence of transcripts mostly related to fermentation pathways. It is interesting to note that Duru et al. (2018) discussed that different bacterial species can be introduced to cheese microbiota at the steps of brining or from contact with processing equipment. These findings point out to the importance of unraveling genetic determinants expressed under processing conditions that favor bacterial persistence in-house, as proposed in this study. According to the literature, *L. lactis* is able to form biofilms, and this property has been linked to the presence of functional pili and mucus-binding proteins that withstand external forces (Oxaran et al., 2012; Drame et al., 2021). Indeed, LAB can form stable biofilms in several types of surfaces, such as glass, stainless steel, and polystyrene (Gómez et al., 2016; Kim et al., 2022), and the formation of biofilms in wooden vats may even contribute to the processing of traditional cheeses (Cruciata et al., 2018). It is interesting to note that in this study with metatranscriptomic datasets from cheese samples from various sources, the transcripts of adhesin genes were the most abundant, and fimbriae (or pili) are adhesins found on the bacterial surface as a complex molecular structure (Abraham et al., 2015).

For a better understanding of the parameters that control cheese ripening, De Filippis et al. (2016) (bioproject #Gs0117939) studied the structure of microbial communities (16S rRNA amplicon) and gene expression profiles (metatranscriptomics) during the processing of a typical medium-ripened pasta filata Italian cheese. Those authors reported that cheese ripening was driven by a few non-starter lactobacilli, which were more abundant in cheese core compared to the crust. It was interesting to note Non-Starter LAB (NSLAB) increased in relative abundance both during cheese maturation and with ripening temperatures. *Lactobacillus fermentum* appeared only at the highest ripening temperature tested, and continued to increase after 10 days. Moreover, De Filippis et al. (2016) reported that ripening-associated microbial metabolism was greater in the cheese core, increased with temperature and it was consistent with aroma profiles. With respect to DESeq analysis, those authors reported 651 genes were differentially expressed depending on the ripening temperature (16°C or 20°C), with over-expression of genes related to protein catabolism/transport and beta oxidation of fatty acids at the highest temperature. Metatranscriptomic and metabolomic data consistently suggested NSLAB are fundamental players in cheese maturation, reinforcing the microbiome can be promptly modified by processing conditions.

Taking into account in-house microorganisms may eventually be found in finished dairy products, irrespective of the use of starter cultures, this research carried out *in silico* with the aim of finding biofilm effectors in dairy-related bioprojects is very relevant for food safety and quality.

Microorganisms in biofilms exhibit coordinated behavior of cells governed by specific regulatory mechanisms, even mimicking the properties characteristic of more conventional multicellular life forms (Penesyan et al., 2021). In this sense, it is of great interest to determine which genes related to biofilm regulatory pathways are expressed under diverse conditions, including those occurring in food processing environments.

Bacteria present a distinct repertoire of signaling components, mainly the TCS based on a conserved phosphotransfer pathway between a HK and a RR protein, where HK catalyzes autophosphorylation at a conserved histidine residue and often possess phosphatase activity toward cognate phosphorylated RR, which is a key switch to control phosphorylation-activated output responses (Gao et al., 2007).

With regard to the RR gene families that were more deeply investigated in this research, the DNA-binding RR retrieved were those encoded by the genes *Amir, ArsR,CsrA, HxlR, LysR, MerR, NarL, NtrC, ompR*, *PrrA, XRE, YcbB,* and *YesN*, out of which many have been implicated with biofilm formation (Redfern et al., 2021). Likewise, genes from the families *CsrA,* and *MerR* have already been directly linked to the formation of biofilms by *E. coli* and *L monocytogenes,* respectively (Jackson et al., 2002; Huang et al., 2012).

The RR *CheV* contains a protein-binding domain, while *CheA, CheB, CheC, CheY, PglZ, PilB*, *PleD, RpfG, TrxB, and WcaA* are regulators that present an enzymatic effector domain. Xu et al. (2021) reported that *CheA, CheB, CheV,* and *CheY* genes, besides being linked to chemotaxis function, may also be crucial for adhesion.

In this study it was also evaluated the possible correlations among the classes of effectors, finding positive correlations for flagellum, RR, HK, adhesins and LuxS. The interaction between HK and chemical signaling molecules activates TCS *via* RR. It has been reported in literature that mutations in some RR genes (for example *degU*that encodes the RR DegU) prevent both flagellum formation and floating biofilm (pellicle) in *Bacillus subtilis*, a bacterium commonly found in soil (Kobayashi, 2007). Similarly, it was demonstrated that for *L. monocytogenes*, DegU is required for flagellar synthesis, motility, virulence, and biofilm formation (Gueriri et al., 2008). TCS are also involved in the expression of flagellum, adhesion, and biofilm-associated genes in *P. aeruginosa* (Prüß, 2017; Zhou et al., 2021). The positive correlation observed for LuxS and flagellum, RR, HK, and adhesins is in accordance with literature, since the LuxS/AI-2 QS system modulates several cellular processes involved in the regulation of virulence factors, including motility and biofilm formation (Wang et al., 2019; Zhang et al., 2019).

On the other hand, no correlations were observed for BAP and AHL, AHL-acylase, adhesin, LuxS, flagellum, or RR. BAP are of key importance for the step of bacterial adhesion, and the interference with BAP expression is expected to diminish biofilm formation (Cucarella et al., 2001; Jordan et al., 2008). There was absence of correlations either for AHL and adhesins or between AHL-acylase versus HK or adhesins. In the literature there are many papers describing a complex interrelationship of virulence factors and QS, besides the influence of environmental factors (i.e., iron concentration), which corroborates the necessity for further investigation about the microbial interactions in biofilms (Azizi et al., 2016).

Overall, the outcome of this research corroborated the hypothesis about the presence of biofilm-related transcripts in datasets from dairy environments, nonetheless the limitations inherent to *in silico* projects (i.e., limited number of samples from each category, samples originated from diverse regions). The knowledge on the expression of protein effectors in dairy datasets is important since these molecules are key for biofilm regulation, and can interfere with adhesion, maturation, dispersal, motility, secretion of exopolysaccharides, and extracellular DNA (Wolska et al., 2016). Disclosing novel regulatory pathways helps in the definition of potential targets to be aimed by combinations of traditional cleaning and sanitizing procedures, as well as with sustainable biological-based products and nanostructured delivery systems.

Finally, the presence of biofilm-related effectors in public metatranscriptomics datasets from dairy-associated samples indicates the possibility of determining specific gene markers to propose mechanisms to interfere with biofilm formation and contribute to avoid persistent microbial contamination in dairies.

## Data availability statement

Publicly available datasets were analyzed in this study. This data can be found at: https://gold.jgi.doe.gov/ and https://www.ncbi.nlm.nih.gov/.

## Author contributions

OA and EM: study concept and design. OA: bioinformatics analysis. OA, EM, and VA: data interpretation and discussion. MP, OA, EM, and VA: drafting of the manuscript. EM, VO, and VA: critical revision of the manuscript for important intellectual content. EM and VA: writing the final version of the manuscript. All authors contributed to the article and approved the submitted version.

## Conflict of interest

The authors declare that the research was conducted in the absence of any commercial or financial relationships that could be construed as a potential conflict of interest.

The reviewer CG declared a shared affiliation with one of the author VA to the handling editor.

## Publisher’s note

All claims expressed in this article are solely those of the authors and do not necessarily represent those of their affiliated organizations, or those of the publisher, the editors and the reviewers. Any product that may be evaluated in this article, or claim that may be made by its manufacturer, is not guaranteed or endorsed by the publisher.

## Footnotes

^1^ https://gold.jgi.doe.gov/

^2^ https://www.ncbi.nlm.nih.gov/
